# Utility of Analgesic and Anxiolytic Medication Dose during Colonoscopy in Identifying Patients with Irritable Bowel Syndrome

**DOI:** 10.5402/2012/969015

**Published:** 2012-01-04

**Authors:** Enoch Lule, Erika Iddings, Lochana Manandhar, Bala Grandhi, John Clements

**Affiliations:** ^1^Medical Center Of Central Georgia, 777 Hemlock Street, Macon, GA 31201, USA; ^2^Valley Gastroenterology Associates, 4860 McLeod Dr Ste A, Saginaw, MI 48604, USA; ^3^Synergy Medical Education Alliance, 1000 Houghton Avenue, Saginaw, MI 48602, USA; ^4^Synergy Medical Education Alliance, 1000 Houghton Avenue, Saginaw, MI 40602, USA

## Abstract

*Research question*. This paper was done to answer the question on whether patients with IBS require higher analgesic or anxiolytic doses during colonoscopy. *Setting*. Gastroenterology practice in Michigan, USA. *Methods*. We reviewed the charts of patients following up with a US based gastroenterology practice. We collected data on whether or not they had IBS, and collected data on analgesic and anxiolytic requirement during colonoscopy. *Results*. 336 patients were included in the trial. 206 did not have IBS while 130 had a previous diagnosis of IBS. 234 were female (67.2%). When comparing patients who have IBS to those without IBS, we identified no statistically significant difference in midazolam dose (5.5 mg versus 5.5 mg), fentanyl dose ( 117 mg versus 112 mg) or meperidine dose (69 mg versus 69 mg). The lack of differences in medication doses used remained when we controlled for sex, prior analgesic use, and prior abdominal surgery. *Conclusion*. Dose of analgesic or anxiolytic used during colonoscopy cannot be used to identify patients with IBS.

## 1. Background

Irritable bowel syndrome (IBS) is a functional GI disorder characterized by abdominal pain and altered bowel habits. Extensive testing during workup for IBS is not recommended [1]. However colonoscopies are commonly done in the diagnostic workup of patients with IBS. A 2005 review of colonoscopies in the US showed that 23.8% were done for IBS symptoms [2]. It is also of note that there is no difference in prevalence of colonic organic lesions when patients with IBS are compared to those without IBS [3]. One study noted that small intestinal biopsy in adults with IBS-like symptoms revealed microscopic lesions in 81% of patients [4].

Visceral hypersensitivity has been proposed as a mechanism of IBS [5]. Some endoscopists also believe that the patients who remain uncomfortable or require increasing doses of analgesics and anesthesia have IBS. In our literature search we found no literature documenting increased analgesic or anxiolytic doses during colonoscopy of patients with IBS.

## 2. Justification

It is unknown whether patients with IBS require higher analgesic doses during colonoscopy. Documentation of increased analgesic or anxiolytic doses may be used to diagnose IBS during colonoscopy, and it may make for safer colonoscopies for patients with IBS as physicians may use higher doses at the onset of colonoscopy.

## 3. Method and Procedures

We carried out a retrospective chart review of patients following with a four physician gastroenterology group that practices in Saginaw, Michigan. Charts were reviewed in alphabetic order for eligibility and included if they met our study criteria.

We included into the study charts of patients who were above the age of 18 years who had undergone colonoscopy. We recorded age, sex, analgesia use prior to colonoscopy, previous abdominal surgery, analgesia dose at time of colonoscopy, and anxiolytic dose at time of colonoscopy.

We also recorded information on indication for colonoscopy and postprocedure diagnosis after colonoscopy. BMI, renal function, and hepatic function within 9 months of the colonoscopy were recorded if it was available.

We excluded patients who underwent emergency colonoscopy, patients who were hemodynamically unstable at the time of colonoscopy, and patients who were on continuous IV analgesic drips or on continuous topical analgesic patches at the time of colonoscopy. Patients who underwent more than one procedure, for example, esophagogastroscopy and colonoscopy, were also excluded. Patients undergoing any procedure other than polypectomy were also excluded. Patients who primarily received any analgesic other than meperidine and fentanyl as the primary analgesic were also excluded.

### 3.1. Statistical Analysis

The dose of analgesic required in IBS patients undergoing colonoscopy was compared to dose of analgesic required in non-IBS patients. The dose of benzodiazepine required during colonoscopy in IBS patients was compared to dose of sedative in non-IBS patients.

All parameters were compared using 2 sided Student *t* tests and considered statistically significant if a *P* value of less than 0.05 was achieved. We then analyzed the data to check if the prespecified potential confounders (age, previous abdominal surgery, prior analgesic use, BMI) had influenced the results.

### 3.2. Sample Size Calculation

Using

- power 80%,
- alpha 0.05,
- versed dose SD range 2 to 7 mg,
- meperidine dose SD range 12.5 to 50 mg,
- with the intention of using comparison by 2 sample independent, *t* test a sample size of 87 was calculated for each group.

### 3.3. Ethical Considerations

The study received IRB approval from Synergy Medical Education Alliance. No personal identifying data was collected.

## 4. Results

We reviewed 1260 charts for eligibility and selected 336 for analysis. We had 234 female and 102 male. The indication for colonoscopy, gross diagnosis, type of previous abdominal surgery, renal function, and liver function was also recorded, and this is given in a supplementary text (see supplementary Material available online at doi:10.5402/2011/969015).

Of the patient charts selected, seen 206 had no previous diagnosis of IBS. 130 had a previous diagnosis of IBS.

81.3% of IBS patients were female whereas 61.2% of non-IBS patients were female. This was a statistically significant difference (Pearson chi square 0.000).

Patients with IBS were also more likely to have undergone previous abdominal surgery (60.8% versus 46.3%, Pearson chi square = 0.010).

When the two groups were compared, patients with IBS were statistically significantly younger (46.4 years versus 53.4 years old, *P* < 0.05).

We had valid BMI data (recordings of weight and height in the 9 months of the colonoscopy) on 87 patients with IBS and 90 patients without IBS. Mean BMI was 28.4 in the IBS group versus 31.6 in the non-IBS group (*t* test *P* = 0.003).

Patients in both groups had statistically similar amounts of opiate and non-opiate prior analgesic use (Pearson Chi Square = 0.216) (see supplement).

On comparing patients with IBS to those without IBS, we found that the patients used similar doses of meperidine (69.3 mg versus 69.4 mg), fentanyl (117.4 mg versus 112.3 mg), or midazolam (5.5 mg versus 5.5 mg) during colonoscopy.

We had a few patients who required additional propofol when undergoing colonoscopy (Table 2). Patients with IBS and those without IBS were statistically as likely as each other to require further analgesia with propofol.

We then carried out sub analysis to see if the lack of difference in analgesic or anxiolytic dose was influenced by the differences in the two groups.

We excluded males and looked at analgesic and anxiolytic doses used only in females. We found no statistically significant differences in doses of medication used (Table 3).

We had found that IBS patients had more previous abdominal surgeries than non-IBS patients. We however did not find any differences in analgesic or anxiolytic doses used in patient who had gone previous surgery versus those who had not.

Though there were statistically significant differences in age (see Table 1) and BMI (BMI of 28.4 in patients with IBS, 31.6 in patients without IBS), we did not feel that these differences were clinically significant.

## 5. Discussion

In this paper, patients with IBS were not more likely to require higher analgesia or anxiolytic medication doses during colonoscopy when compared with the general population of other patients undergoing colonoscopy.

The lack of difference could not be explained by group differences in sex, age, previous abdominal surgery, previous analgesic use, or BMI in the comparison groups.

We were able to achieve the predetermined sample size for analysis of comparison of meperidine and midazolam doses used in the two groups.

Our study had the same percentage of females identified with IBS and following with a physician (81.3%) as a previous 2005 survey [6]. The average age of patients with IBS in our trial (46.4 years) may have been older than other studies because our population was people undergoing colonoscopy and this might have caused a selection bias that skewed the mean age upwards.

We looked at previous abdominal surgery as a potential confounder because we thought that patients who have had abdominal surgery may form increased adhesions, and this would tether their colons and make them require more analgesia. This turned out not to be the case (Table 4).

In light of the previous studies on hypersensitivity as a mechanism of IBS, the most likely explanation for lack of difference in medication requirement is the possibility that initial doses requested by the gastroenterologist were sufficient to cause analgesia and relaxation in the same percent of IBS patients and non-IBS patients.

We also note that previous studies showing hypersensitivity in IBS were done comparing patients with IBS to patients to the normal population [5] while in our study population we found that 66.7% of patients had identifiable gross pathology on colonoscopy (see supplement).

We however note some limitations in our study. Analgesia and anxiolytic doses were done at the discretion of the gastroenterologists, without a formal protocol. We also note that there may have been patients with undiagnosed IBS in the non-IBS group. In a general population survey, this percentage of individuals was found to be about 10.8% [6]. We however expect the number in this study to be much lower as all patients were following up with a gastroenterology practice.

In conclusion, our study found no relationship between analgesic and anxiolytic dose during colonoscopy and the presence of IBS. We therefore note that medication dosing during colonoscopy cannot be used by physicians to suspect IBS and medication for colonoscopy of patients with IBS should be the same as for the general population undergoing colonoscopy.

## Supplementary Material

The supplement first details indications of colonoscopy. Where more than one indication of colonoscopy is given, it is listed as indication 2 and indication 3 if necessary. The table headed MISC shows infrequently given indications for colonoscopy. The tables on gross diagnosis indicate the Gastroenterologists recorded findings on colonoscopy. If more than one pathology was identified it is listed as gross diagnosis 2. The tables on abdominal surgery indicate whether the patient had recorded surgery prior to colonoscopy and what that surgery was. The final table indicates prior analgesic use in patients with IBS compared to those without IBS.

## Figures and Tables

**Table 1:** Age, meperidine, fentanyl, and midazolam dose in patients with IBS compared to patients without IBS.

	IBS	number	mean dose	std deviation	sig (2 tailed)
Meperidine	No	129	69.44	25.243	
	Yes	94	69.28	23.594	0.962
Fentanyl	No	77	112.33	37.252	
	Yes	36	117.36	30.366	0.449
Midazolam	No	206	5.510	1.9044	
	Yes	130	5.485	2.1404	0.913

**Table 2:** Patients requiring propofol in addition to meperidine or fentanyl.

	No propofol	Propofol
IBS	125	5
No IBS	204	2

Pearson Uncorrected 3.230 (*P* = 0.072.).

**Table 3:** Female patients' meperidine, fentanyl and midazolam dose when comparing patients without IBS to patients with IBS.

		*N*	MEAN	STD DEVIATION	Sig (2 tailed)
Meperidine	NO IBS	78	69.49	24.81	0.850
	IBS	78	68.75	23.64	
Fentamyl	NO IBS	48	114.05	43.04	0.673
	IBS	30	117.50	28.73	
Midazolam	NO IBS	126	5.68	1.99	0.663
	IBS	108	5.57	2.11	

*N*: number, Std deviation: Standard deviation.

**Table 4:** Meperidine, fentanyl and midazolam dose in patients who have had abdominal surgery compared to those who have not had abdominal surgery.

		*N*	Mean	Standard deviation	Sig (2 tailed)
Meperidine	No surgery Abd surgery	112	69.75	24.410	0.750
		110	68.70	24.644	
Fentanyl	No surgery Abd surgery	49	112.76	37.542	0.760
		64	114. 84	33.473	
Midazolam	No surgery Abd surgery	161	5.612	1.941	0.332
		174	5.339	2.051	

(Abd: abdominal, *N*: Number).
